# PROTOCOL: Effectiveness of economic development interventions in humanitarian settings in low‐ and middle‐income countries: A mixed‐method systematic review

**DOI:** 10.1002/cl2.1357

**Published:** 2023-10-19

**Authors:** Suchi Kapoor Malhotra, Marcella Vigneri, Nina Ashley O. Dela Cruz, Heather MacDonald, Howard White

**Affiliations:** ^1^ Campbell South Asia New Delhi India; ^2^ Global Development Network New Delhi India; ^3^ London School of Hygiene and Tropical Medicine London UK; ^4^ Campbell Collaboration New Delhi India; ^5^ Carleton University Ottawa Canada; ^6^ Evidence‐Based Public Policy Lanzhou University Gansu China

## Abstract

This is the protocol for a Campbell systematic review. The review will address the following research questions: (1) What are the effects of economic development interventions on the economic outcomes of people in humanitarian settings? What factors explain any observed variations in effect such as setting, programme design features or duration? (2) What are the effects of economic development interventions on the food security, nutrition, the psychosocial and mental health, and the physical health outcomes of populations in humanitarian settings? (3) What are the success factors and barriers that affect the implementation and effectiveness of economic development interventions on populations affected by humanitarian crisis?

## 1 BACKGROUND

### 1.1 The problem, condition or issue

#### 1.1.1 The issue

*Humanitarian crises* affect communities and people across the world, causing high levels of mortality or malnutrition, the spread of disease and epidemics and health emergencies, and arresting economic growth. Several causes may trigger a humanitarian crisis: political events such as armed conflicts, coups, ethnic and religious persecution, and environmental catastrophes such as floods, earthquakes, and typhoons.

##### Environmental causes

Exposure to natural hazards has rapidly increased over the past decades due to ecological degradation and climate change (Benevolenza & Derigne, 2019; Guha‐Sapir et al., 2012). The World Meteorological Organization states that weather‐related disasters have increased fivefold over the last 50 years.^1^ These disasters have severe local and regional effects on the populations affected. Natural disasters often cause tremendous socioeconomic losses to human communities (van den Berg, 2010; Thurston et al., 2021). Women and children often face a disproportionate burden during and after the crises (United Nations Office for Disaster Risk Reduction, 2011). Natural disasters in the year 2020 alone affected approximately 100 million people and this has estimated that 190 billion US$ of global economic losses and it also resulted in 15,082 deaths (Jones et al., 2022).

##### Political unrest and conflict

In 2020 there were 56 active armed conflicts around the world, with more than 50 active conflicts every year since 2015 (Strand & Håvard, 2021). By mid‐2020 over 100 million people around the world were displaced by persecution and conflict, most of whom were displaced within their home country (UNHCR, 2022).

##### Population displacement

According to UN High Commissioner for Refugees, there were 82.4 million forcibly displaced people in the world at the end of 2020 out of which nearly a third were refugees (UNHCR, 2022). Refugees and internally displaced populations (IDPs) that are affected by natural disasters and political unrest require accommodation, housing, and key public services such as health care and education, and will at some point seek to provide for their livelihood. They will look for work in the informal or formal labour market and interact economically with the host economy in multiple ways. The impact of forcibly displaced persons on residents’ livelihoods in host communities is a serious challenge, especially in developing countries with limited financial and administrative capacities. When developing countries host refugees, they sometimes receive short‐term financial and technical support from the international community. But most refugee populations remain in their new location for years rather than weeks and months. Hence relying on relief not only is unsustainable, but also fails to draw on the skills of the refugee population (Harrell‐Bond, 1986; Schneiderheinze & Lücke, 2020). Since populations affected by humanitarian crises can remain displaced for a protracted period, they need assistance in acquiring skills, training and economic opportunities that can lead to self‐reliance and develop new livelihoods to rebuild lives in the aftermath of the crisis (Devictor, 2019).

Refugees bear the risk of rising debt levels and asset depletion, and families face indebtedness to cover the cost of shelter and other basic needs. A survey of Syrian refugees staying in Lebanon reported that 90% borrow money or receive credit, and this leads them to the risk of debt bondage (Bermudez, 2017).

This review focuses on post‐emergency economic interventions that provide economic opportunities to populations affected by humanitarian crisis to transition from relief to sustainable development. In particular, the focus is on economic interventions such as livelihood support programmes that hold the potential of restoring economic independence, dignity, and self‐reliance.

Importantly, the presence of a large number of populations displaced either by conflict or natural disasters in their countries of origin, also represents an economic shock to the host economy once refugees begin to interact with residents on a large scale. Managing economic interactions between refugees and residents is a necessary condition to ensure that refugees can live with dignity and integrate economically and socially into the host community. Investing in the economic development of the area in which refugees are settled provides benefits to the host population, who may otherwise become resentful when they see the scale of services passing them by to benefit the influx of refugees (Fajth et al., 2019; Harrell‐Bond, 1986).

#### 1.1.2 Humanitarian settings

The review assesses economic development interventions in humanitarian settings. The settings considered include natural disasters (broadly classified as biologic, climate‐related, or geophysical) and political unrest and armed conflicts. We include both populations forced to move from their homes to escape disaster‐driven devastation and political violence, and those remaining in place post‐disaster. In these settings, new economic opportunities are a means to restore livelihoods and to facilitate the creation of new integrated communities.

The review includes economic development interventions such as livelihoods programmes, market support programmes, and local area development projects. Economic development interventions support economic development in the area in which humanitarian emergencies occur. We focus on interventions and programmes that aim to bridge the transition from emergency response to the development of local economic systems post‐conflict and post‐disaster in low‐ and middle‐income countries (Leaning & Guha‐Sapir, 2013).

The review does not include the impact of humanitarian interventions in general, and it is not all economic interventions. We exclude cash transfers because they are mainly used to support consumption, not production.^2^ However, we include cash transfers delivered in conjunction with economic development interventions. Other eligible evaluations include economic development interventions and livelihood programs serving refugees, internally displaced persons (IDPs) from natural disasters that are intended to lead to sustainable employment and income‐generating activities. Similarly, we consider interventions that sustain the recovery and development of refugees and internally displaced people, which account for local market demands and build on existing skills and experience of the target population (Jacobsen & Fratzke, 2016). We also include empowerment programmes that have an economic component (e.g., savings clubs and microcredit).

Populations affected by humanitarian crises require special assistance in developing skills, training and economic opportunities that can lead to self‐reliance and develop new livelihoods to rebuild lives in the aftermath of the crisis (Devictor, 2019). To do this effectively, programs must be designed to meet the specific needs of the target community. For example, where refugee camps for post‐conflict displaced people are built in areas with limited economic opportunity and underdeveloped infrastructure, it is essential to establish effective foundations by creating important infrastructure and services in education, WASH, nutrition, and shelter (Als et al., 2020). Likewise, natural disasters’ induce disruption to both built and natural environments. In post‐disaster settings interventions to promote livelihood development need to include investments in infrastructure such as news roads, refurbished markets, processing facilities and micro credit schemes that are necessary to initiate and sustain income generating activities (Sina et al., 2019).

The novelty of this review is the inclusion of economic development interventions in humanitarian crisis, both those affected by conflict and those affected by natural disasters. Although these settings involve different populations, different political contexts, and different development possibilities, the populations affected share several features in terms of vulnerable groups, needs, skillset to develop in the aftermath of a crisis and in how they respond to different inputs and activities from the interventions that are targeting sustainable economic development.

### 1.2 How the intervention might work

To improve the effectiveness of economic development interventions, attention needs to be given to the design of programmes that promote livelihoods beyond the aftermath of the crisis, and that put project affected populations on a sustainable growth path. Several such approaches have been developed, such as market support programmes, savings schemes, job training programmes, women's collective action groups to economically empower smallholders that face additional gender bias constraints in the aftermath of a crisis (Buvinic et al., 2013).

It is not always clear which of these approaches is the most effective in relation to a specific humanitarian setting. Because this review cuts across humanitarian settings and different populations, it is useful to understand how interventions generate sustainable impacts. To do this, we build a causal process Theory of Change (ToC), also referred to as middle‐level theory (MLT). MLT is a conceptual framework (Cartwright et al., 2020) to identify causal pathways to impact which are transferable across different humanitarian settings and draw from the existing evidence a clear understanding of which interventions result in long‐term, sustainable livelihoods outcomes.

The MLT approach is particularly appropriate for systematic reviews that look at different settings and populations to identify and test the assumptions under which different specific interventions generate the intended outcomes. Rather than looking at whether specific interventions work in different categories of humanitarian settings, the MLT approach identifies and tests common, transferable causal pathways to impact by exploring and testing which assumptions hold (White, 2018).

**Figure 1:**
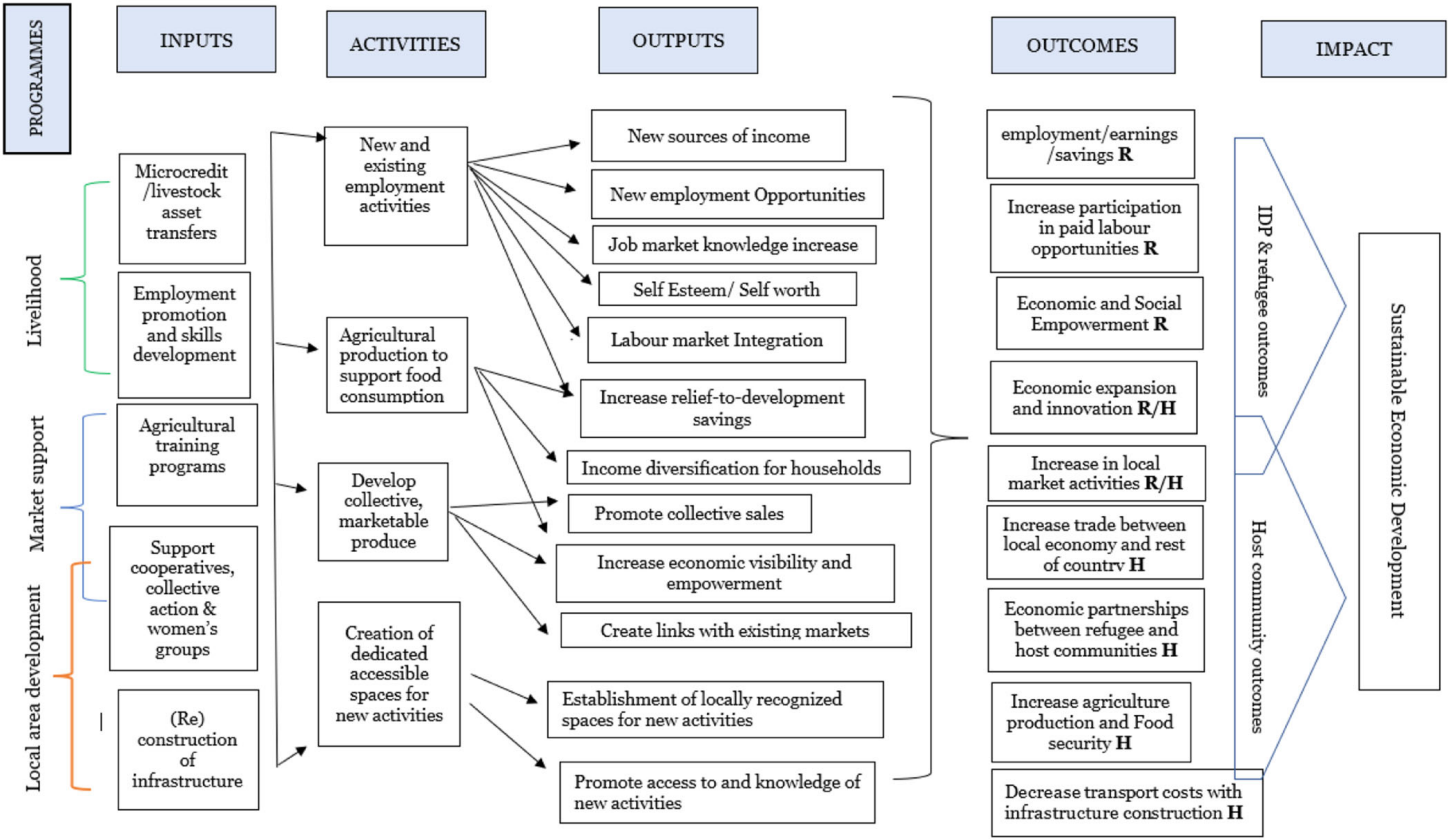
General theory of change for economic interventions in humanitarian setting: A livelihood framework. **R** indicates intended outcomes for Refugees (both post‐conflict and natural disaster affected populations). **H** indicates outcomes that are likely to affect the Host populations.

Figure 1 presents a high‐level representation of the causal pathways of different economic development interventions that may be implemented in humanitarian settings. This high‐level approach helps to identify common causal pathways for different post‐crisis situations. Evidence regarding common causal pathways can generate transferable knowledge about what works to promote sustainable development in humanitarian settings. This may get overlooked by researchers and policymakers specialised in just one type of humanitarian crisis. The visual presentation below shows the causal process through which inputs (interventions) are turned into activities (implemented for post‐crisis and post‐disaster populations), outputs (precursors of economic activities), final outcomes (potential income generating opportunities), and impact (sustainable economic development).

The left side of the figure lists the economic development interventions that may be relevant across different humanitarian settings:

- Agricultural training programmes,
- Microcredit schemes and asset (e.g., livestock) transfers,
- Employment promotion and skills development schemes,
- Support to cooperatives and collective action groups,
- Construction and rebuilding of physical and environmental infrastructure,

These interventions are followed by the activities they intend to promote. Each causal pathway in Figure 1 can be traced from left to right. To illustrate this, we take the example of interventions focusing on skills development and employment promotion (Habiyakare et al., 2015). These interventions support the acquisition of market‐relevant skills, which should lead to new employment activities that increase the sources of income. The activities may be associated with increased integration into local markets. These outcomes may lead to sustained economic development.

Our approach is based on the livelihood's framework, developed by Scoones (1998), which has been applied in humanitarian settings by both researchers—see an early literature review by Longley and Maxwell (2003) and a recent study of Syrian refugees in Lebanon (Al Zoubi et al., 2019)—and practitioners—for example, Catholic Relief Service's Guidance on Livelihoods Programming in Emergency Response and Recover Contexts (Catholic Relief Services, 2018). Central to the livelihood's framework is the asset framework which includes all assets contemplated in our framework: financial capital is provided through microfinance interventions, human capital through training, and physical capital through infrastructure. Natural, political, and social capital appear in the assumptions as elements which are required for interventions to work.

It is not always clear which of these approaches is the most effective in relation to humanitarian crisis settings where evaluations of programmes tend to focus on intended outcomes and impacts (whether the intervention worked and what effect it had on outcomes) but but not on assessing the process of implementation and on establishing how the use of a specific approach leads to changes in outcomes (Ruaudel & Morrison‐Métois, 2017). Because this review cuts across humanitarian settings and different displaced populations, understanding which interventions generate impacts that are achievable, sustainable, and transferable is critical.

#### 1.2.1 Assumptions

Here we list the assumptions underpinning the theory of change. These are not features that we assume to hold, but conditions that need to be true for the intervention to work as intended along the causal pathways.

Several conditions (assumptions) need to be in place for the intervention to operate through the pathway to impact. For example: refugees and IDPs often struggle to integrate in the host labour market due to the *loss of assets and separation from family members* (Schuettler & Caron, 2020). The *lack of skills* required in the local labour market and the *absence of social networks* may act as discriminating factor that inhibit access to the labour market at destination (Steimel, 2017). Therefore, the success of employment promotion and development skills programmes depends on the assumptions that an assessment of the demand and supply side of the labour market, including the legal situation of those forcibly displaced and their perceptions and aspirations, has taken place before designing interventions (Verrinder & Kamash, 2019).

Micro‐finance or other form of loans are a means to support agricultural production or other small business. Populations affected by humanitarian crisis, however, face additional difficulties to access credit due to the perceived temporariness of their stay, lack of collateral and the associated perceived higher risk of non‐repayment. The success of these schemes may depend on the ability to obtain agricultural land for farming, or on the purchase of productive assets to build business skills, both necessary assumptions for the effectiveness of microcredit schemes. Graduation‐type programs, for example, are a clear example of how these schemes may work for the extreme poor and for vulnerable populations to progress into sustainable livelihoods: combining cash grants to build up assets with entrepreneurship training, and intensive coaching and financial inclusion (Schuettler & Caron, 2020).

To take one more example, in forced displacement contexts, vocational, business, and other skills programs aim to overcome the mismatch between the skills of those forcibly displaced and the needs of the host labour market to be appropriately organised (World Bank, 2017). If, for example, refugees report struggling to find time to attend training programs even if they believe they would benefit from them, or if social or cultural norms (such as norms that discourage women from appearing in public) are a barrier to attendance, the programme needs to be designed with greater flexibility in the organization of the trainings. Thinking through the assumptions thus provides ideas on programme design.

We note that Figure 1 represents a visual illustration of causal pathways of impact from possible intervention types to outcomes and impact. While there will be intermediary outcomes to consider on a case by case that lead to different avenues of impact, we maintain a visual representation of the ToC at a higher level and return to a discussion of detailed pathways to impact in the findings section.

We also acknowledge here the possibility that economic interventions might work differently for refugee/IDP populations affected by climatic shocks and for those affected by violent conflict. The adverse consequences associated with climate change—water scarcity, crop failure, food insecurity, economic shocks, migration, and displacement‐ can act as a threat both in the immediate and long term by intensifying conflict over scarce resources, reducing economic opportunities and social cohesion, as well as straining public institutions and trust in the state. When people are forced to move away from their homes, they lose their land, jobs, homes, and access to food, setting the stage for more fragility and instability. The range of possible economicinterventions that can promote recovery may include the creation of migration corridors, shared water points, surveillance for major diseases, as well as strengthened early warning systems and enhanced crises response. Other interventions may focus on helping communities stay in place where local adaptation options are viable, while also helping people move away from unavoidable climate risks.

In conflict‐affected populations, displacement can exacerbate inequalities and the potential for further conflict, especially in areas that have limited access to services and few economic opportunities. In these instances, inclusive policies and development investments for both those who have been forcibly displaced and for host communities, can mitigate the negative effects of displacement and foster social cohesion. Economic interventions that promote sustainable development may include progressive policies that grant refugees and IDPs the right to work, freedom of movement, access to social services, as well as property. These interventions serve the important purpose of promoting social and economic development, which is in turn a pre‐condition for the economic recovery and sustainable growth.

#### 1.2.2 Assumptions

**Table:** 

Availability of facilities for training	Market knowledge for the timely sale of produce	Mobility of refugees and IDPs
Accessible and context appropriate content of training	Access to natural capital or other assets required for productive activities	Attitudes towards investment in new job opportunities
Knowledge of local needs and trading practices for (re)construction of infrastructure	Promotion of job opportunities through adequate communication	Improved physical and mental health post‐disaster
	Advertisement of new skilled workforce to facilitate integration in the labour market	
	Social capital for procurement and marketing channels	
	No political obstacles to new businesses, e.g., licensing requirements and harassment by authorities	

### 1.3 Why it is important to do the review

The review aims to address several evidence gaps in the literature on specific aspects of economics development interventions. We identified four gaps which this review could contribute to. First, an existing review by Juillard et al. (2017) ‘The Influence of Market Support Interventions on Household Food Security: An evidence synthesis, has a limited outcome focus than this proposed review which encompasses household economy more broadly.

Second, Carter, (2016) ‘Economic and market resilience before and aftershocks’ rapid review of humanitarian and disaster risk reduction (DRR) market support interventions, looks at reinforcing economic resilience following natural disasters and conflict. The review is limited to market support programmes.

Third, Brody and co‐authors’ systematic review (2015) examine the impact of women's economic self‐help groups (SHGs) on women's individual‐level empowerment in low‐ and middle‐income, and the opportunities of empowerment that result from participation in economic SHGs. The review, however, does not focus on studies in humanitarian settings.

Fourth, the systematic review by Lwamba et al. (2021) summarises the evidence on the effect of gender‐specific and gender‐transformative interventions on women's empowerment and gender equality in fragile and conflict‐affected states. The review assesses whether the interventions contribute to inclusive and sustainable peace but does not focus on the long‐term economic growth and livelihood opportunities of the different types of interventions considered.

## 2 OBJECTIVES

The review will address the following research questions:

1. What are the effects of economic development interventions on the economic outcomes of people in humanitarian settings? What factors explain any observed variations in effect such as setting, programme design features or duration?
2. What are the effects of economic development interventions on the food security and nutrition, the psychosocial and mental health, and the physical health outcomes of populations in humanitarian settings?
3. What are the success factors and barriers that affect the implementation and effectiveness of economic development interventions on populations affected by humanitarian crisis?

## 3 METHODS

### 3.1 Criteria for considering studies for this review

The studies included in the review will meet the following selection criteria for the programs that evaluate economic development interventions in humanitarian crisis settings. We will include only those studies that are published in English language and there is no exclusion based on the publication year.

These criteria are elaborated below.

#### 3.1.1 Types of participants

People in low‐ and middle‐income countries living in conflict‐affected and natural disaster settings, and populations displaced as a result of both humanitarian crisis. We will also include host populations. We will refer to all these groups as populations affected by humanitarian crisis. We will only include studies where all participants in the study meet the above inclusion criteria.

#### 3.1.2 Types of interventions

Interventions that foster economic development in humanitarian and refugee settings. These include livelihoods programmes, market support programmes, and local area development projects. Also included are interventions that focus on empowerment and include an economic component (such as savings clubs and microcredit schemes).

Examples of eligible interventions are in the following studies:

1. Čelebić (2014) evaluated the effects of the Revitalizing Agricultural/Pastoral Incomes and New Markets (RAIN) programme. The program's goal was to increase the resiliency of households, communities, and market systems to prepare for, cope with and recover from external shocks.
2. Hussam et al. (2021) evaluated the benefits of employment opportunities for the Rohingya refugees of Myanmar.
3. Blattman and Annan (2016) evaluated the effect of Action on Armed Violence (AoAV) intensive agricultural training program on employment activities, income, and socio‐political integration in Liberia.
4. Adoho et al. (2014) evaluated the first round of the EPAG (‘Economic Empowerment of Adolescent Girls and Young Women’) skills training program implemented in post‐conflict Liberia. EPAG was designed to alleviate the barriers to entering the labour market faced by young women.
5. Glass et al. (2017) evaluated the effectiveness of a hybrid microcredit/livestock asset transfer programme—Pigs for Peace (PFP)—on economic, health, and intimate partner violence (IPV) outcomes in post‐conflict settings.

#### 3.1.3 Types of outcome measures

Primary outcomes considered in this review include economic outcomes (income, employment, livelihood, poverty, farm, and business net income). Secondary (intermediate) outcomes include food security and nutrition, social (including attitudes to refugees) and language skills, and physical and mental health.

Table 1 gives examples of the primary and secondary outcomes considered in this review.

**Table 1:** Outcome categories, with examples of each outcome.

Outcome category	Examples
Economic outcomes	Income, poverty, employment, earnings, and savings
	Economic empowerment, economic stability (e.g., livestock/animal assets, reduced credit), economic recovery, market system
Food security and nutrition	Food security (e.g., dietary diversity, macro and micronutrient intake), child nutritional status.
Mental health and psycho‐social health	Mental health (anxiety, depression, and stress) psycho‐social health (self‐esteem and self‐worth; psychosocial well‐being, self‐confidence, investment behaviour; attitudes to refugee populations; social cohesion.)
Physical health	Physical health (morbidity, mortality)
Others	Language skills

We will include all studies where either the primary or secondary outcomes are relevant for the review.

#### 3.1.4 Types of studies

This is a mixed methods review that will include different study designs to address our research questions (RQ). To evaluate the effectiveness of the economic interventions (RQ 1 & 2), we will include:

∘ Experimental designs: randomised controlled trials (RCTs).
∘ Non‐experimental designs: Designs with a non‐randomly assigned comparison group, or regression designs which control for selection bias (instrumental variables, regression discontinuity, and Heckmann model). Difference‐in‐difference analysis is included if either (1) the parallel trends assumption is satisfied, or (2) a statistical matching procedure is used to create the comparison group.

We will not include Before and After studies with no comparison group to ensure that impact is measured across groups that are equally eligible to receive the intervention, but only one of the groups is exposed to the intervention. Studies with both active and passive controls will be included. We will not include economic evaluations or cost‐effectiveness studies.

To understand the success factors and possible barriers to participation in the economic interventions (RQ 3) we will include:

∘ Process evaluations and qualitative studies of interventions: any evaluation or study of an eligible intervention discussing design and implementation issues.
∘ Information on barriers and facilitators will also be extracted from effectiveness studies if reported.

### 3.2 Search strategy

#### 3.2.1 Electronic searches

We will use the following strategies to identify completed and on‐going potential studies:

##### Database and platform

- Scopus,
- Web of Science core collection,
- CABI platform‐ World Agricultural Economics and Rural Sociology Abstracts, CAB Abstract,
- Ebscohost platform—Econlit, CINAHL
- Proquest Platform—PAIS, Worldwide Political Science Abstracts, ERIC, PsycINFO, ASSIA, Social Services Abstracts
- Web of Science platform—Medline

Supporting Information: Appendix A presents an example of the search strings used for publication databases and search engines, with terms for interventions, regions, and methodologies.

#### 3.2.2 Searching other resources

In addition to searching electronic databases, we will also screen the bibliographies of included studies and existing reviews of humanitarian intervention programmes for eligible studies. A full list of list of research organisations and websites that we will search for any relevant publications is provided in Appendix B.

### 3.3 DATA collection and analysis

#### 3.3.1 Selection of studies

The screening of studies for inclusion/exclusion will be undertaken in two stages using EPPI reviewer 4. The first stage is the title and abstract screening, and the second stage is the full text screening. The first stage of title and abstract screening will be assisted by priority screening, which is the machine learning function in EPPI. Both stages of screening will be done by two independent researchers (SM and MV) using the screening tool, with a third‐party (HW) arbitrating in case of disagreement. The screening tool is included in the Supporting Information in Appendix C.

#### 3.3.2 Data extraction and management

For impact and process evaluations/qualitative studies, we will use a standardised data extraction form (Supporting Information: Appendix D) to extract data from all the studies that meet our inclusion criteria. Data extraction from each study will include context/geographical information, population, study design and method, intervention types and outcomes type, and subcategories. Three researchers (SM/MV/NA) will conduct the data extraction for each study. All three coders have been trained on the tool before starting. Disagreements will be resolved through discussion with a third reviewer (HW) consulted as needed.

#### 3.3.3 Assessment of risk of bias in included studies

The critical appraisal tool will help reviewers provide an indication of the quality of the confidence of the findings included in the review. The confidence in the findings of all studies included in the review will be assessed using a risk of bias tool for the effectiveness studies, and a critical appraisal tool for the process evaluation and qualitative studies developed by the Campbell Collaboration Secretariat. See Appendix E for a version of the tool. Coding for critical appraisal will be carried out by two independent reviewers (SM/NA) with a third person (HW) arbitrating any discordance.

### 3.4 Analysis and presentation

#### 3.4.1 Unit of analysis issues

In this review, the unit‐of‐analysis for the included studies is individual women or men and households receiving exposure to the programme. Studies will usually report data as averages at the programme level, both for all people/families in the programme, and possibly for sub‐groups categorised by age, sex, and location.

#### 3.4.2 Criteria for determination of independent findings

Multiple papers or reports based on the same study or data will be treated as single case.The report or the paper will only be treated as a separate case if the study sample does not include study participants included in any other coded study. If there are multiple versions of the same paper then the most recent version will be used, unless an older version provides estimates not available in the later version.

All relevant effect sizes will be coded and there will be multiple effect sizes for an outcome from a single study. For multiple measures of the same outcome or sub‐outcome, we will use a three‐level meta‐analytical model to estimate weighted mean effect sizes for all outcomes to account for the dependency between effect sizes (Viechtbauer, 2010). This approach can incorporate the inclusion of multiple effect sizes from the same evaluations for different outcomes (Hedges et al., 2010).

#### 3.4.3 Dealing with missing data

Study authors will be contacted if we require additional data that is missing or incomplete in the paper. In case of no‐availability/no response from authors, the studies with missing or incomplete data will be exluded from the meta‐analysis.

#### 3.4.4 Statistical procedures and conventions

Outcomes reported as binary variables (e.g., gender) will be converted into odds ratios via derivation of a 2 × 2 table from the reported results (e.g. percentages will be converted to absolute numbers by multiplying by the sample size).

To ensure the comparability of effect size across different evaluations, we will transform effect size estimates into odds ratios using the natural logarithm of the ratio. Then the hedges g will be derived from Cohen's d (Polanin & Snilstveit, 2016).

For continuous outcome variables we will calculate Hedge's g (as Hedge's *g* is preferred over Cohen's for small samples as we anticipate will be the case for this review). Each study will be checked to ensure that outcomes are consistently coded.

A meta‐analysis for each outcome will be conducted using Stata. As per the recommendation of Moeyaert et al. (2017), a three‐level meta‐analytical model will be used if an adequate number of studies are identified (i.e., 50 or more studies). If fewer studies are identified, then a robust variance estimation will be used to account for the dependence between effects (Moeyaert et al., 2017).

A moderator analysis of the effect size for a single categorical variable will be conducted using a subgroup analysis, also using a random‐effects model. Meta‐regressions (*metareg* command in Stata) that account for moderating factors such as study design, intervention characteristics, length of programme, and country income will be used to supplement the analysis.

#### 3.4.5 Assessment of heterogeneity

Heterogeneity between effect sizes across studies included will be assessed by reporting the Q‐value, degrees of freedom and the value of *I* ^2^. Forest plots will be generated for the visual representation of pooled effect size on both economic outcomes and social outcomes. The causes of heterogeneity, if any, will be identified by visual inspection and by a moderator analysis (Table 2). Separate forest plots will be presented for important moderators.

**Table 2:** Moderators from the theory of change analysis.

Characteristic	Moderators
Participant characteristics	Female/male/mixed
	Young women
	Women with disabilities
Scale	Local, regional or national
Location	Rural, urban or both
Duration of intervention	Days, weeks or months
Intensity	Activity/session duration
	Activity/session frequency
Design of the programme	Savings schemes
	Job training programmes
	women's collective action groups
Type of settings	School, community, other
	Humanitarian setting—Natural disaster
	Armed conflict
Type of population	Refugees/internally displaced persons (IDPs)
	Humanitarian/natural disaster affected
	Conflict affected populations
Age group	Age ranges
Sex	All males
	All female
	Mixed
	Not known
Ethnicity	All or predominately minority ethnic group (80%+)
	Substantial minority ethnic group (30%–79%)
	No or minority of minority ethnic group (<30%)
Type of study design	Experimental
	Non‐experimental
	Process evaluation
Confidence in study findings	High confidence
	Medium confidence
	Low confidence
Time of effect measurement	Endline
	Up to 6 months
	7–18 months
	19–35 months
	36 months or more

#### 3.4.6 Treatment of publication bias

Publication‐selection bias will be assessed for the primary economic outcomes by constructing funnel plots for each of the outcomes (Higgins & Green, 2009). Funnel plots will be used for a trim‐and‐fill analysis and for the calculation of Egger's test.

#### 3.4.7 Planned moderator analyses

Table 2 summarises the moderators suggested by our theory of change (Table 1 and Figure 1), as well as moderators based on demographic characteristics.

In addition, we will also include as moderators (i) the region of the intervention (South Asia, Sub‐ Saharan Africa, etc)., (ii) publication type (i.e., published vs. unpublished); (iii) study design, and (iv) confidence in study findings (risk of bias). We will compute a multilevel meta‐regression in STATA to compute mean effect sizes for each subgroup and compare the nature of the differences between subgroups.

Post hoc moderator analyses may be used depending on the analysis of patterns of heterogeneity in the data.

#### 3.4.8 Sensitivity analysis

Sensitivity analysis will be carried out by removing studies from the meta‐analysis one‐by‐one to see if the results of the meta‐analysis are sensitive to any single study. We will also conduct a sensitivity of findings by risk of bias (low risk, some concerns, and high risk).

#### 3.4.9 Mixed method analysis (treatment of qualitative research)

This review adopts mixed‐method approach that combines qualitative data with a quantitative meta‐analysis, within the framework of a theory‐based systematic review, TBSR (White, 2018). The TBSR approach, which has similarities with the framework synthesis approach (Booth & Carroll, 2015; Carroll, et al., 2013), takes the intervention as the unit of analysis, not the individual study. Different studies may contribute findings at different stages of the causal chain. For example, process evaluations and qualitative studies shed more light on implementation issues, whereas effectiveness studies explain both the size of, and variations in, effects.

The TBSR framework is shown in Table 3. Quantitative data are indicated as Qt and qualitative as Ql. Quantitative data refers to both effect sizes and factual quantitative data such as participation rates.

**Table 3:** Stages of the causal chain with data to be examined at each stage.

Stage in causal chain	Data
Awareness of the programme amongst relevant service providers and target group	Knowledge of programme, aware of eligibility criteria, purpose and how to access (Qt/Ql)
Activities undertaken	Descriptive material (Ql)
Connection to services	Channels for service connection (Ql)
Design of the programme	Savings schemes, job training programmes, women's collective action groups (Qt/Ql)
Economic outcomes	Income, employment, livelihood, poverty, farm or business net income. (Qt supported by Ql).
Social (includes attitudes)	Self‐esteem and self‐worth; Psychosocial wellbeing, Self‐confidence, Investment behaviour; Host attitudes to refugee populations; social cohesion (Qt supported by QI)

Table 3 shows the TBSR framework which is used for both horizontal and vertical synthesis (White, 2018). In Table 4 an abbreviated version of the row headings from Table 1 is pivoted to become column headings. The data in Table 3 are subject to vertical, horizontal, and total synthesis.

**Table 4:** Theory‐based systematic framework.

	Participation	Activities	Programme design	Services	Economical	Social	
Case 1							Horizontal synthesis
Case 2							
‐‐‐							
Case n							
	Vertical synthesis						Overall synthesis

Vertical synthesis involves summarising the evidence across all cases, which is the way systematic reviews are usually performed, especially for quantitative analysis of effects. In the case of qualitative data, vertical synthesis is a thematic analysis, in which common themes are identified across studies.

Horizontal synthesis summarises across a case—which may be done in narrative reviews, but with the difference here that the data for an intervention may come from more than one study.

The overall synthesis combines both, though it may contain separate overall synthesis by sub‐group (e.g., for populations affected by natural disasters and for populations affected by conflict). The overall synthesis approach, drawing on both horizontal and vertical synthesis, ‘tells the story’: whether the intervention works, for whom, under what circumstances, and why.

## ROLES AND RESPONSIBILITIES

- Suchi Kapoor Malhotra: Project lead responsible for project management, report writing, search, and screening and coding and statistical analysis and meta‐analysis.
- Marcella Vigneri: Lead for Statistical analysis and meta‐analysis, also screening and coding, and writing and reviewing outputs.
- Heather MacDonald: Information specialist, helped in systematic searching of the databases.
- Nina Ashley O. Dela Cruz: Screening, coding and statistical analysis and meta‐analysis.
- Howard White: Technical and strategic support for conducting the review and will provide overall intellectual direction for the review.

## POTENTIAL CONFLICTS OF INTEREST STATEMENT

No conflict of interest.

## PRELIMINARY TIMEFRAME

Note, if the protocol or review is not submitted within 6 months and 18 months of title registration, respectively, the review area is opened up for other authors.

- Date you plan to submit a draft protocol: May 2022
- Date you plan to submit a draft review: September 2022

## Supporting information

Supporting information.

## Footnotes

1
https://public.wmo.int/en/media/press-release/weather-related-disasters-increase-over-past-50-years-causing-more-damage-fewer

2 In principle, cash transfers, rather than in‐kind, mean that refugees will use local shops, and so stimulate the local economy rather than bypass it (or even flood it with resales of in‐kind donations), but this aspect is not considered in cash transfer impact evaluations.
