# Supplementary material for: PROTOCOL: Effectiveness of economic development interventions in humanitarian settings in low‐ and middle‐income countries: A mixed‐method systematic review
Source: Campbell Syst Rev. 2023 Oct 19;19(4):e1357. doi: 10.1002/cl2.1357 (PMC10585609; doi:10.1002/cl2.1357)
Supplement: Supplementary file 1 — Supporting information. [file CL2-19-e1357-s001.docx]

**Appendix A**

**Search string**

| **SCOPUS** |  |  |  |
| --- | --- | --- | --- |
| **Search string** | **Results** |  |  |
| TITLE-ABS-KEY(conflict* OR postconflict* OR disaster* OR postdisaster* OR crisis OR crises OR displaced OR displacement OR refugee* OR asylum-seeker* OR war OR armed-intervention OR flood* OR cyclone* OR earthquake* OR tsunami* OR tidal-wave* OR volcan* OR hurricane* OR typhoon* OR avalanche* OR drought* OR famine OR starvation OR landslide* OR rockslide* OR mudslide* OR catastrophe OR genocide OR epidemic OR after-shock* OR aftershock* OR external-shock*) | 3,136,777 |  |  |
| TITLE-ABS-KEY((humanitar* OR ((job OR agricultur* OR farm* OR vocational ) W/1 (training)) OR employment* OR skill-development OR skills-development OR reconstruct* OR rebuild* OR livelihood* OR economic-development OR (develop* W/1 econom*) OR economic-opportunit* OR economic-empower* OR savings-club* OR savings-schem* OR micro-finance OR microfinance OR micro-credit OR microcredit OR micro-enterprise OR microenterprise OR property-right* OR (relief W/1 development) OR “empower* program*” OR self-help-group* OR ((support* OR program* OR group*) W/3 (cooperative* OR collective*)) OR ((women* OR girl*) W/2 (collective* OR empower*)) OR ((market*) W/0 (support* OR system* OR service* OR infrastructure OR labor OR labour)) OR ((livestock OR asset*) W/1 (transfer*))) W/9 (intervention* OR approach* OR framework OR theory-building OR theoretical OR conceptual OR Initiative* OR assistance OR platform* OR project* OR program* OR policy OR policies OR action*)) | 187,312 |  |  |
| TITLE-ABS-KEY((impact W/1 evaluat*) OR ((program*) W/5 (evaluat* OR impact* OR assess*)) OR (project* W/5 evaluat*) OR process-evaluation OR "random* control* trial*" OR "random* trial*" OR rct* OR (random* W/3 allocat*) OR (random* W/1 evaluat*) OR clinical-trial OR equivalence-trial OR double-blind OR single-blind OR "instrumental variable*" OR "synthetic control" OR intervention-stud* OR (experimental W/1 (study OR design OR evaluat*)) OR quasi-experiment* OR dif-dif OR "double difference" OR difference-in-difference OR "difference in difference" OR "multiple regression" OR "multivariate regression" OR "multivariable regression" OR "bivariate regression" OR "statistical regression" OR "regression discontinuity*" OR “regression analysis” OR "statistical matching*" OR "propensity score matching" OR "covariate matching" OR "coarsened-exact matching" OR "propensity-weighted" OR matched-pair OR mixed-method* OR meta-analysis OR cohort-stud* OR cross-sectional OR cohort-analysis OR case-control OR retrospective-stud* OR retrospective-evaluation OR follow-up-stud* OR longitudinal-stud* OR prospective-stud* OR epidemiologic* OR cross-over-stud* OR quantitative-method* OR interrupted-time-series OR ( before W/5 after ) OR ( pre W/5 post ) OR ( ( pretest OR pre-test ) AND ( posttest OR post-test ) ) OR case-stud* OR case-report OR ( fixed-effect* W/3 ( model OR estimation ) ) OR ( random-effect* W/3 ( model OR estimation ) ) OR (bivariate AND model) OR (multivariate AND model) OR ((quantitative OR comparison-group* OR counterfactual OR counter-factual OR experiment* OR comparative ) W/3 ( design OR study OR analysis)) OR ((semi-structured OR semistructured OR prestructured OR pre-structured OR unstructured OR informal OR in-depth OR indepth OR face-to-face OR structured OR guide) W/2 (interview* OR discussion* OR questionnaire* OR survey)) OR focus-group* OR qualitative OR ethnograph* OR fieldwork OR field-work OR field-experiment OR key-informant OR participatory OR action-research OR cooperative-inquiry OR co-operative-inquiry OR community-led OR barrier* OR facilitator* OR enabler*) | | 21,540,329 | |
| TITLE-ABS-KEY(afghanistan OR albania OR algeria OR "american samoa" OR angola OR "antigua and barbuda" OR antigua OR barbuda OR argentina OR armenia OR armenian OR aruba OR azerbaijan OR bahrain OR bangladesh OR barbados OR belarus OR byelarus OR belorussia OR byelorussian OR belize OR "british honduras" OR benin OR dahomey OR bhutan OR bolivia OR "bosnia and herzegovina" OR bosnia OR herzegovina OR botswana OR bechuanaland OR brazil OR brasil OR bulgaria OR "burkina faso" OR "burkina fasso" OR "upper volta" OR burundi OR urundi OR "cabo verde" OR "cape verde" OR cambodia OR kampuchea OR "khmer republic" OR cameroon OR cameron OR cameroun OR "central african republic" OR "ubangi shari" OR chad OR chile OR china OR colombia OR comoros OR "comoro islands" OR "iles comores" OR mayotte OR "democratic republic of the congo" OR "democratic republic congo" OR congo OR zaire OR "costa rica" OR "cote d’ivoire" OR "cote d’ ivoire" OR "cote divoire" OR "cote d ivoire" OR "ivory coast" OR croatia OR cuba OR cyprus OR "czech republic" OR czechoslovakia OR djibouti OR "french somaliland" OR dominica OR "dominican republic" OR ecuador OR egypt OR "united arab republic" OR "el salvador" OR "equatorial guinea" OR "spanish guinea" OR eritrea OR estonia OR eswatini OR swaziland OR ethiopia OR fiji OR gabon OR "gabonese republic" OR gambia OR "georgia (republic)" OR georgian OR ghana OR "gold coast" OR gibraltar OR greece OR grenada OR guam OR guatemala OR guinea OR "guinea bissau" OR guyana OR "british guiana" OR haiti OR hispaniola OR honduras OR hungary OR india OR indonesia OR timor OR iran OR iraq OR "isle of man" OR jamaica OR jordan OR kazakhstan OR kazakh OR kenya OR "democratic people’s republic of korea" OR "republic of korea" OR "north korea" OR "south korea" OR korea OR kosovo OR kyrgyzstan OR kirghizia OR kirgizstan OR "kyrgyz republic" OR kirghiz OR laos OR "lao pdr" OR "lao people's democratic republic" OR latvia OR lebanon OR "lebanese republic" OR lesotho OR basutoland OR liberia OR libya OR "libyan arab jamahiriya" OR lithuania OR macau OR macao OR "macedonia (republic)" OR macedonia OR madagascar OR "malagasy republic" OR malawi OR nyasaland OR malaysia OR "malay federation" OR "malaya federation" OR maldives OR "indian ocean islands" OR "indian ocean" OR mali OR malta OR micronesia OR "federated states of micronesia" OR kiribati OR "marshall islands" OR nauru OR "northern mariana islands" OR palau OR tuvalu OR mauritania OR mauritius OR mexico OR moldova OR moldovian OR mongolia OR montenegro OR morocco OR ifni OR mozambique OR "portuguese east africa" OR myanmar OR burma OR namibia OR nepal OR "netherlands antilles" OR nicaragua OR niger OR nigeria OR oman OR muscat OR pakistan OR panama OR "papua new guinea" OR "new guinea" OR paraguay OR peru OR philippines OR philipines OR phillipines OR phillippines OR poland OR "polish people's republic" OR portugal OR "portuguese republic" OR "puerto rico" OR romania OR russia OR "russian federation" OR ussr OR "soviet union" OR "union of soviet socialist republics" OR rwanda OR ruanda OR samoa OR "pacific islands" OR polynesia OR "samoan islands" OR "navigator island" OR "navigator islands" OR "sao tome and principe" OR "saudi arabia" OR senegal OR serbia OR seychelles OR "sierra leone" OR slovakia OR "slovak republic" OR slovenia OR melanesia OR "solomon island" OR "solomon islands" OR "norfolk island" OR "norfolk islands" OR somali* OR "south africa" OR "south sudan" OR "sri lanka" OR ceylon OR "saint kitts and nevis" OR "st. kitts and nevis" OR "saint lucia" OR "st. lucia" OR "saint vincent and the grenadines" OR "saint vincent" OR "st. vincent" OR grenadines OR sudan OR suriname OR surinam OR "dutch guiana" OR "netherlands guiana" OR syria OR "syrian arab republic" OR tajikistan OR tadjikistan OR tadzhikistan OR tadzhik OR tanzania OR tanganyika OR thailand OR siam OR "timor leste" OR "east timor" OR togo OR "togolese republic" OR tonga OR "trinidad and tobago" OR trinidad OR tobago OR tunisia OR turkey OR "turkey (republic)" OR turkmenistan OR turkmen OR uganda OR ukraine OR uruguay OR uzbekistan OR uzbek OR vanuatu OR "new hebrides" OR venezuela OR vietnam OR "viet nam" OR "middle east" OR "west bank" OR gaza OR palestine OR yemen OR yugoslavia OR zambia OR zimbabwe OR "northern rhodesia" OR "global south" OR "africa south of the sahara" OR "sub-saharan africa" OR "subsaharan africa" OR "africa, central" OR "central africa" OR "africa, northern" OR "north africa" OR "northern africa" OR magreb OR maghrib OR sahara OR "africa, southern" OR "southern africa" OR "africa, eastern" OR "east africa" OR "eastern africa" OR "africa, western" OR "west africa" OR "western africa" OR "west indies" OR "indian ocean islands" OR caribbean OR "central america" OR "latin america" OR "south and central america" OR "south america" OR "asia, central" OR "central asia" OR "asia, northern" OR "north asia" OR "northern asia" OR "asia, southeastern" OR "southeastern asia" OR "south eastern asia" OR "southeast asia" OR "south east asia" OR "asia, western" OR "western asia" OR "europe, eastern" OR "east europe" OR "eastern europe" OR "developing country" OR "developing countries" OR "developing nation*" OR "developing population*" OR "developing world" OR "less developed countr*" OR "less developed nation*" OR "less developed population*" OR "less developed world" OR "lesser developed countr*" OR "lesser developed nation*" OR "lesser developed population*" OR "lesser developed world" OR "under developed countr*" OR "under developed nation*" OR "under developed population*" OR "under developed world" OR "underdeveloped countr*" OR "underdeveloped nation*" OR "underdeveloped population*" OR "underdeveloped world" OR "middle income countr*" OR "middle income nation*" OR "middle income population*" OR "low income countr*" OR "low income nation*" OR "low income population*" OR "lower income countr*" OR "lower income nation*" OR "lower income population*" OR "underserved countr*" OR "underserved nation*" OR "underserved population*" OR "underserved world" OR "under served countr*" OR "under served nation*" OR "under served population*" OR "under served world" OR "deprived countr*" OR "deprived nation*" OR "deprived population*" OR "deprived world" OR "poor countr*" OR "poor nation*" OR "poor population*" OR "poor world" OR "poorer countr*" OR "poorer nation*" OR "poorer population*" OR "poorer world" OR "developing econom*" OR "less developed econom*" OR "lesser developed econom*" OR "under developed econom*" OR "underdeveloped econom*" OR "middle income econom*" OR "low income econom*" OR "lower income econom*" OR "low gdp" OR "low gnp" OR "low gross domestic" OR "low gross national" OR "lower gdp" OR "lower gnp" OR "lower gross domestic" OR "lower gross national" OR lmic OR lmics OR "third world" OR "lami countr*" OR "transitional countr*" OR "emerging econom*" OR "emerging nation*") | | 6,503,928 | |
| 1 AND 2 AND 3 AND 4 | | 4,562 | |

**Appendix B – List of websites to be hand-searched**

**List of research organizations and websites**

- World Bank,
- ILO,
- USAID,
- African Development Bank,
- JPAL,
- IPA,
- FAO,
- Save the children,
- UNICEF,
- 3ie,
- UN Women,
- United Nations Office for the Coordination of Humanitarian Affairs,
- Reliefweb,
- Humanitarian Aid International Inter-American Development Bank (IADB).

**Appendix C Screening tool**

This review evaluates the interventions that foster economic development in humanitarian and refugee settings. These include livelihoods programmes, market support programmes, and local area development projects. Also included are interventions that focus on women's empowerment that include an economic component (such as savings clubs and microcredit schemes).

| Screening tool | | | |
| --- | --- | --- | --- |
| 1. | Is the paper in English? | No | Exclude |
|  |  | Yes | Continue to q2 |
|  |  |  |  |
| 2 | Is the people living in low- middle income countries living in humanitarian and conflict-affected settings? | No | Exclude |
|  |  | Yes | Continue to q3a |
|  |  |  |  |
| 2. | Is the paper about an intervention intended to foster economic development in humanitarian and refugee settings? | No | Exclude |
|  |  | Yes | Continue to q3a |
|  |  |  |  |
| 3a. | Is the paper a quantitative evaluation reporting measures of eligible outcomes compared to the outcomes (1) in a comparison group (either with or without baseline outcome measures). | No | Continue to q3b |
|  |  | Yes | Continue to q4 |
|  |  |  |  |
| 3b. | Is the paper a qualitative process evaluation describing intervention design or implementation? | No  Yes | Exclude  Include (END) |
|  |  |  |  |
| 4. | Do any outcome measuring economic development (Income, Employment ), food security, Nurtition, Social, Phsyical and Mental health. | No | Exclude |
|  |  | Yes | Include |

**Appendix D Coding tools**

| Category | Sub Category |
| --- | --- |
| Publication Status | Ongoing  Completed |
| Region | East Asia & Pacific  Europe & Central Asia  Latin America & Caribbean  Middle East & North Africa  North America  South Asia  Sub Saharan Africa  South America |
| Area | Rural  Urban  Rural and Urban (Both)  Not Clear |
| Ages | Age  Not reported |
| Participant characteristics | Female  Male  Mixed  Young women  Women with disabilities |
| *Types of Populations* | Refugees/Internally displaced persons (IDPs)  Humanitarian/Natural Disaste**r** Affected  Conflict affected populations |
| BAME | Mainly/exclusively (80%)  Partly  None  Not clear |
| Project/ Intervention Name |  |
| Year |  |
| Scale of the programme | Local,  Regional  National |
| *L*evel of intervention | Individual,  Household  Community |
| Type of Settings | School, Community, Other  Humanitarian Setting - Natural Disaster  Armed Conflict |
| Intervention duration | Duration  Not reported |
| Intervention sub-categories | Livelihoods programmes,  Market support programmes,  Local area development projects.  Women's empowerment programmes that include an economic component (such as savings clubs and microcredit schemes |
| Study Design | Experimental design  Non- Experimental design  Process Evaluation |
| Study Method | Randomized Controlled Trial  Non- Experimental effectiveness study  Process evaluation or qualitative intervention study |
| Sample Size | Total Numbers  Total Number of Participants in Intervention Group  Total Number of Participants in Control Group |
| Attrition | Number |
|  |  |
| Outcome Domain | Outcome Sub-domain |
| Economic outcomes | Income, poverty, employment, earnings, and savings  Economic empowerment, Economic stability (eg, livestock/animal assets, reduced credit), Economic recovery, Market system |
| Food Security and nutrition | Food security (e.g. dietary diversity, macro and micro nutrient intake), Child nutritional status |
| Social (including attitudes) | Self-esteem and self-worth; Psychosocial wellbeing, Self- confidence, Investment behaviour; Host attitudes to refugee populations; social cohesion. |
| Physical and mental health | Physical and mental health |
| Others | Language skills |
| Time of effect measurement | Endline  Up to 6 months  7-18 months  19-35 months  36 months or more |
| Barriers and Facilitators to the participation |  |
| Barriers and Facilitators to Outcome |  |
| Design Issues |  |
| Implementation Issues |  |
| What affected populations say |  |
| Moderators and Confounders |  |

**Effect size coding**

| Effect size | Numerical entry |
| --- | --- |
| Outcome name | Open coding |
| Outcome domain | For each outcome coded |
| Type of effect | Difference in means  Difference in proportions  Regression coefficient  Odds ratio  Risk ratio |
| Sub group analysis | Whole sample  Sub-group (name) |
| Duration | Endline  Post endline (duration) |
| Sample sizes | For each effect coded, and corresponding control sample size |
| Standard deviations | For each outcome coded |
| Treatment effect | Intention to treat  Treatment of treated |

**Appendix E: Critical appraisal tool**

The critical appraisal tool helped reviewers provide an indication of the quality of the confidence of the findings included in the review. All studies were rated against how clear the intervention and evaluation questions described in the study and overall scores were also calculated in the same way.

For more a more detailed look at study quality, separate questions were considered for impact and process evaluations because they have different purposes and therefore different elements that can affect their quality.

**Risk of Bias tool for primary studies: effectiveness**

| **Bias domain** | **Question** | **Scoring criteria** | **Decision rules** |
| --- | --- | --- | --- |
| **1** | **1a. Confounding**:  Was the allocation or identification mechanism able to address confounding? |  |  |
|  | - **RCT** | **a) Sequence generation:**  **-** The authors describe a random component in sequence generation/ randomisation method (e.g., lottery, coin toss, random number table).*  **-** If a special randomisation procedure is used to ensure balance, it is well described (stratification, pairwise matching, unique random draw, multiple random draws etc.) and adjustment is considered in the analysis (e.g., stratum fixed effects, pairwise matching variables).  **b) Subversion:**  - if the unit of allocation was by beneficiary or group, there was some form of centralised allocation mechanism such as an on-site computer system to ensure adequate allocation concealment.  **-** If a public lottery was used for the sequence generation, details were given on the exact settings and participants attending the lottery.  **c) Balance:**  **-** The unit of allocation is based on a sufficiently large sample size to equate groups on average.  **-** A balance table is reported for all subgroups receiving differential treatment, comparing means and standard deviations of variables, including cluster-level variables. | - Score “Low risk” if all criterion are satisfied.  - Score "Some concerns" if there is no balance table reported (or key variables are omitted from the table) -- Score "High risk" if there is any failure in the allocation mechanism which could affect the randomisation process, or there is no balance table reported (c) and there is evidence suggesting a problem in the randomisation, such as covariate means are very different or sample size is too small for the procedure used (using stratification when there are less than two units for each intervention and control group in each strata can lead to imbalance), or if the paper does not provide details on the randomisation process or uses quasi-randomisation (e.g., alternate households allocated) which it is not clear has generated allocations equivalent to randomisation.  * In order to assess the validity of the quasi-randomisation process, the most important aspect is whether the assignment process might generate a correlation between participation status and other factors (for example, gender, socio-economic status, pre-existing health condition) determining outcomes; consider whether assignment is done at cluster level (centralised) and covariate balance is reported. |
|  | - **NRS using statistical matching** | **a)** Information about the programme targeting criteria are known, presented in the paper, and used to justify the statistical approach.  **b)** Matching is done on pre-test (or time-invariant) characteristics, including the outcome measured at pre-test; matches are geographically local; the variables used to match are relevant (for example, demographic and socio-economic factors) to explain both participation and the outcome (so that there can be no evident differences across groups in variables that might explain outcomes); and, for cluster-assignment, authors control for external cluster-level factors that might confound the impact of the programme.* **c)** With the exception of Kernel matching, the means of the individual covariates are demonstrated to be equated for treatment and comparison groups after matching. | -Score "Low risk”, if all criteria are addressed. -Score "Some concerns " if the selection into the programme was done according to clear targeting rules, which are used as matching variables, but there are imbalances remaining after matching. -Score "High risk" if programme assignment was self-selected by participants and no baseline data are available to match the participants or groups, or matching was done based on variables that are likely to be affected by the programme, or relevant variables are not included in the matching equation including cluster-level variables. * Accounting for and matching on all relevant characteristics is usually only feasible when the programme allocation rule is known and there are no errors of targeting. There are different ways in which covariates can be considered. Observable differences across groups can be incorporated in the framework of a regression analysis (e.g., propensity-weighted least squares) or can be assessed by testing equality of means between groups. Differences in unobservable characteristics can be account for using double differences (DD), fixed effects (FE) or random effects (RE) where unobservables are time-invariant. |
|  | - **NRS using double differences (DD), fixed effects (FE) or random effects (RE) analysis of panel data*** | **a)** Outcomes are measured at pre-test (before intervention) and post-test (after intervention) using the same approach. **b)** Examination of secular trends in outcomes shows parallel trends across treatment and comparison groups during periods prior to intervention.  **c)** The method is combined by well-conducted statistical matching done according to clear programme allocation rules (see above), and baseline imbalances, including in the outcome are shown to be small.  **d)** A comprehensive set of individual time-varying characteristics is controlled, including any cluster-level covariates that may affect the impact of the programme (e.g., rainfall).** | -Score "Low risk” all criteria are addressed. -Score "Some concerns" if selection into the programme was done according to clear rules, and equal trends demonstrated, but baseline imbalances between groups remained. -Score "High risk " if equal trends are not reported, and programme allocation was due to participant self-selection, programme allocation was self-selected by participants and some relevant time-varying characteristics are not controlled, or insufficient details are provided, for example on testing the equal trends assumption or about cluster-level variables.  * DD, FE and RE regression models are sometimes complemented with matching strategies. This combination approach is superior since it only uses in the estimation the common support region of the sample size, reducing the likelihood of existence of time-varying unobservable differences across groups affecting outcome of interest and removing biases arising from time-invariant unobservable characteristics.  ** Knowing allocation rules for the programme – or even whether the non-participants were individuals that refused to participate in the programme, as opposed to individuals that were not given the opportunity to participate in the programme – can help in the assessment of whether the covariates accounted for in the regression capture all the relevant characteristics that explain differences between treatment and comparison. |
|  | **1b. Confounding - justification** | Justification for coding decision (include a brief summary of justification for rating, mentioning your response to all sub-questions, cite relevant pages). | |
| **2** | **2a. Selection bias:** was any differential selection into the study adequately resolved? | **a) Follow-up data:** If the study design is prospective, follow-ups are recorded for all eligible participant units from recruitment onwards (i.e., prior to treatment). This is best shown using a participant flow diagram or reporting sufficient information to construct one.  **b) Participant identification:** where the unit of allocation in a prospective study was at group level (geographical/ social/ cluster unit), allocation was performed on all units at the start of the study, or participants and recruiters are blinded to allocation status, or awareness is unlikely to affect recruitment differentially (e.g., participants chosen randomly using a sampling frame based on census and response rate is high).  **c) Balance:** a table is reported for all subgroups receiving differential treatment within control or treatment groups, comparing means and standard deviations of variables; any unbalanced covariates at individual level are controlled in adjusted analysis, including cluster-level variables.  **d) Selection bias analysis:** where evidence suggests there is selection bias into the study due to censoring of data (e.g., immortal time bias), this is accounted for using appropriate statistical methods (e.g., propensity weighted regression, Heckman selection model, proportional hazards model). | -Score “Low risk” if all relevant criteria are satisfied.  -Score “Some concerns” if the study used prospective design with adequate concealment, but no (or an incomplete) study flow diagram is reported, or in retrospective design where statistical methods are used to correct for selection bias.  -Score “High risk” if there are threats to adequate concealment (e.g., individual participants were chosen after cluster assignment was conducted or known, and there are differences between characteristics of the two groups beyond those expected by chance alone), or there is evidence of differential recruitment into study arms and differences in characteristics of groups not compatible with chance, or if no information is presented about participant characteristics or, in a prospective study, no study flow diagram (or data to construct it) presented. |
|  | **2b. Selection bias - justification** | Justification for coding decision (include a brief summary of justification for rating, mentioning your response to all sub-questions, cite relevant pages). | |
| **3** | **3a. Attrition bias:**  was any differential selection out of the study adequately resolved? | **a) Attrition at cluster-level:** is sufficiently low and similar reasons for attrition in treatment and control. Sufficiently low attrition is defined as:  **-** total attrition (losses to follow-up) between pre-test and post-test in the study less than 10 percent of clusters (low risk) or 20 percent (some concerns).  **-** differential cluster attrition across study arms is less than 10 percentage points, and reasons for attrition are given and similar across groups.  **b) Attrition at individual-level:** is sufficiently low and similar reasons for attrition in treatment and control. Sufficiently low attrition is defined as:  **-** total attrition (losses to follow-up) between pre-test and post-test in the study less than 10 percent of observations (low risk) or 20 percent (some concerns).  **-** differential attrition across study arms is less than 10 percentage points, and reasons for attrition are given and similar across groups.  **c) Robustness to attrition:** the study assesses losses to follow-up to be random draws from the sample (for example, by examining correlation with key characteristics across groups, or an F-test of attrition on baseline characteristics and interacted with treatment status), and study participants are randomly sampled. | -Score "Low risk" if overall attrition is less than 10 percent and differential attrition less than 10 percentage points at cluster (a) and individual (b) levels, and the study demonstrates robustness to attrition.  -Score "Some concerns" if overall attrition is between 10% and 20% and differential attrition less than 10 percentage points.  -Score "High risk" if overall attrition exceeds 20% or differential attrition exceeds 10 percentage points, or there is some indication that the survey respondents were purposively sampled in a way that might have led the sampling to be different between treatment and control groups, or there is insufficient information on sampling methods, or no information on attrition is given. |
|  | **3b. Attrition bias - justification** | Justification for coding decision (include a brief summary of justification for rating, mentioning your response to all sub questions, cite relevant pages). | |
| **4** | **4a. Motivation bias:** was the process of observation free from motivation bias? | **Are criteria adequately addressed?**  **a)** For data collected in the context of a particular intervention trial (randomised or non-randomised assignment), the authors state explicitly that the process of monitoring the intervention and outcome measurement is blinded to participants and outcome assessors, or methods are used that would minimise risk of Hawthorne effects, John Henry effects or survey effects such as infrequent observation or outcome questionnaires not referring to the intervention. Authors may also adapt the study design to estimate possible survey and Hawthorne effects (e.g., a ‘pure control’ with no monitoring except baseline endline). **b)** Informed consent is not associated with a particular intervention, as in the case of a regular household survey or a cluster-RCT, data are collected from administrative records, or in the context of a retrospective (*ex post*) evaluation. | -Score “Low risk” if either criterion is satisfied. -Score "Some concerns" if there was imbalance in the frequency of monitoring in intervention groups, which could have influenced behaviour in treatment and control differentially. - Score "High risk" if authors do not use an appropriate method to prevent possible motivation biases through blinding or other controls (e.g., infrequent measurement, methods to ensure consistent monitoring across groups, measurement using a ‘pure control’). |
|  | **4b. Motivation bias - justification** | Justification for coding decision (include a brief summary of justification for rating, mentioning your response to all sub questions, cite relevant pages). | |
| **5** | **5a. Performance bias:**  was the study adequately protected against spillovers, no-shows and crossovers? | **a)** There were no implementation issues that might have led the control participants to receive the treatment, or authors use intention-to-treat (ITT) estimation.  **b)** The intervention is unlikely to spill over to comparisons (e.g., participants and non-participants are geographically and/or socially separated from one another and general equilibrium effects are not likely), or the potential effects of spillovers were measured (e.g., variation in the % of units within a cluster receiving the treatment).  **c)** There is no risk of substitution (differential contamination) by external programs (also called treatment confounding): participants are isolated from other interventions which might be received differentially between treatment and controls which could explain changes in outcomes.  **d)** Errors in implementation fidelity by the intervening body were not systematic, or unlikely to affect the outcome.  **e)** For continuous interventions, measurement is taken of adherence to treatment among participants. | -Score “Low risk” if all criteria are satisfied.  -Score "Some concerns" if there is no obvious problem but there is no information reported on potential risks related to spillovers or contamination in the control group, or if there were issues with spillovers but they were controlled for or measured, or if any of the criteria are not satisfied but the scale of the issue is minimal.  -Score “High risk” if any of the criterion are not satisfied and happened at a large scale in the study, or if spillovers, no-shows, crossovers, implementation fidelity, or adherence to continuous interventions, are not reported clearly. |
|  | **5b. Deviation from interventions -justification** | Justification for coding decision (include a brief summary of justification for rating, mentioning your response to all sub questions, cite relevant pages). | |
| **6** | **6a. Measurement error:**  is the study free from biases in measurement of intervention and outcomes? | **a)** The study is a prospective design or in a retrospective design, participation in the intervention is observed, or the intervention clearly and consistently defined and misreporting by participants or enumerators is unlikely.  **b)** Outcomes are clearly and consistently defined for all participants and outcome assessors in the study.  **c)** Outcomes are measured through observation (rather than self-report), and outcome assessors are blinded to intervention or it is shown they are unbiased (e.g., spot-checks to validate).  **d)** For self-reported outcomes: respondents in the intervention group are not more likely to report accurately than controls due to recall bias.  **e)** Respondents do not have incentives to over/under report something related to their performance or actions, or researchers put in place mechanisms to reduce the risk of reporting bias (irregular or infrequent data collection rounds, outcome assessors not involved in the implementation of the intervention, it is clear that answers to the survey will not affect what they receive in the future), or authors have measured bias through falsification tests (e.g., ‘placebo outcomes’ in cases where there was a risk of reporting bias).  **f)** Timing of the data collection did not differ between intervention and comparison group, the baseline data are not likely to be differentially affected by the time of intervention (e.g., due to seasonality). | -Score “Low risk” if all criteria are satisfied.  -Score "Some concerns" if there is a small risk related to any criteria and potential biases are measured, e.g., with placebo outcomes, and found to be null.  -Score "high risk" if there are risks related to any criteria and authors were not able to control for the bias, or no information is provided to justify the absence of bias. |
|  | **6b. Measurement error - justification** | Justification for coding decision (include a brief summary of justification for rating, mentioning your response to all sub questions, cite relevant pages). | |
| **7** | **7a.Analysis reporting bias: RCTs**  Was the study free from selective analysis reporting? | **a)** Authors report results corresponding to the outcomes announced in the method section (there is no outcome reporting bias).  **b)** Authors report multiple analyses appropriately (e.g., by age group, sex).  **c)** A pre-analysis plan or trial protocol is published and referred to or the trial was pre-registered, or the outcomes were pre-registered.  **d)** Authors report appropriate analysis methods, including results of unadjusted analysis and ITT estimation, alongside any adjusted and treatment-on-the-treated/complier-average-causal-effects analysis.  **e)** Analysts were blinded to treatment status. | -Score "Low risk" if all criteria are satisfied.  -Score "Some concerns" if all the conditions are met except a), or if all the conditions are met but there is some element missing that could have helped understand the results better.  -Score "High risk" if no pre-analysis plan or trial protocol was published or pre-registered. |
|  | **7b.Analysis reporting bias: NRS**  Was the study free from selective analysis reporting? | **a)** There is no evidence that outcomes were selectively reported (e.g., results for all relevant outcomes in the methods section are reported in the results section). **b)** Authors use credible methods of analysis to address attribution given available data. **c)** A pre-analysis plan is published, especially for prospective NRS (but ideally also for retrospective studies). **d)** Requirements for specific methods of analysis: - For RDD, Researchers should analyse the change in slope and/or level using different band-widths around the threshold or functional form. The following should be pre-specified as far as possible and reported in sensitivity analysis: (a) selection of optimal bandwidth using existing data-driven routines; (b) selection of appropriate functional form for the relationship between assignment and outcome variables; and (c) robustness checks of other bandwidths and functional form specifications.  - For PSM and covariate matching: (a) Where over 10% of participants fail to be matched, sensitivity analysis is used to re-estimate results using different matching methods (Kernel Matching techniques); (b) For matching with replacement, no single observation in the control group is matched with a large number of observations in the treatment group, and authors take into account the use of control observations multiple times against the same treatment in the standard error calculation; (c) for PSM, Rosenbaum’s test suggests the results are not sensitive to the existence of hidden bias; (d) different matching methods including varying sample sizes yield the same results. - For IV models, the authors test and report the results of a Hausman test for exogeneity (p≤0.05 is required to reject the null hypothesis of exogeneity).  - For Heckman selection models, the coefficient of the selectivity correction term (Rho) is significantly different from zero (p<0.05). | -Score “Low risk” if all criteria are satisfied. -Score "Some concerns" if authors combined methods and reported relevant tests (d) only for one method, or if all the criteria are met except for c) and it is a retrospective NRS. -Score "High risk" if authors use uncommon or less rigorous estimation methods such as failure to conduct multivariate analysis for outcomes equations, or if some important outcomes are subsequently omitted from the results or the significance and magnitude of important outcomes was not assessed. |
|  | **7c. Analysis reporting bias - justification** | Justification for coding decision (include a brief summary of justification for rating, mentioning your response to all sub questions, cite relevant pages). | |

**Critical appraisal tool – Process evaluation**

**Questions for process evaluations (apply to implementation sections) [used for any study coded as having implementation evidence]**

|  |  | High | Medium | Low |  | Low |
| --- | --- | --- | --- | --- | --- | --- |
| 1 | Is the qualitative methodology described? | Yes |  | No | >> 3 |  |
| 2 | Is the qualitatively methodology appropriate to address the evaluation questions? | Yes | Partially | No |  | Insufficient detail |
| 3 | Is the recruitment or sampling strategy described? | Yes |  | No | >> 5 |  |
| 4 | Is the recruitment or sampling strategy appropriate to address the evaluation questions? | Yes | Partially | No |  | Insufficient detail |
| 5 | Are the researcher's own position, assumptions and possible biases outlined? | Yes | Partially | No |  |  |
| 6 | Have ethical considerations been sufficiently considered? | Yes | Partially | No |  | Insufficient detail |
| 7 | Is the data analysis approach adequately described? | Yes |  | No | >>9 |  |
| 8 | Is the data analysis sufficiently rigorous? | Yes | Partially | No |  |  |
| 9 | Are the implications or recommendations clearly based in the evidence from the study? | Yes | Partially | No |  |  |
| 10 | Overall (including questions for all studies- The overall score uses the weakest link in the chain principle i.e., is the lowest score on any item | High: High on all items  Medium: No lower than medium on any item  Low: At least one low |  |  |  |  |

**Appendix F Definitions of outcomes**

| Outcome category | Sub-category | Description |
| --- | --- | --- |
| **Economic Outcomes** (**Economic**) | Employment | Employment measures including job quality |
|  | Income/ Earning and Savings | Earnings and expenditure |
|  | Poverty | Reduction of Poverty |
|  | Economy Stability | Individual/Household ability to access resources essential to life (e.g., livestock/animal assets, house, reduced credit) |
|  | Economic Recovery | Recovery and access to jobs opportunities/ Employment |
|  | Market system | Participation in Market system such as producers, buyers, and consumers. |
|  | Economic Empowerment | Economic empowerment is the capacity of women and men to participate in, contribute to and benefit from growth processes in ways that recognise the value of their contributions, respect their dignity and make it possible to negotiate a fairer distribution of the benefits of growth (OECD definition) |
| *Food Security and Nutrition* | Food Security | (e.g. dietary diversity, macro and micro nutrient intake), |
|  | Child Nutritional Status | Measure of child nutritional status (Anthropometric measurements to assess growth and development,) |
| ***Social Outcomes/ Attitude***  **(Social Outcomes and** An attitude refers to how someone thinks or feels about something whereas a belief is an acceptance that something is true**)** | [Self-esteem / self-worth](about:blank) | Confidence in one’s own worth |
|  | Psychosocial wellbeing | Psychological well-being, self-esteem, self-efficacy, sense of inclusion and entitlement. |
|  | Self – Confidence | Confidence and it is an attitude about the skills and abilities |
|  | Investment behaviour | Behaviour- Risk propensity, risk preference, and attitude |
|  | Host attitudes to refugee populations | An attitude (positive/negative) towards to refugees population |
|  | social cohesion | connectedness and solidarity among groups in society |
| Physical and mental health | Physical health | Any measure of physical health |
|  | Mental health | Any measure of mental health |
